# iPSC-derived mesenchymal stromal cells are less supportive than primary MSCs for co-culture of hematopoietic progenitor cells

**DOI:** 10.1186/s13045-016-0273-2

**Published:** 2016-04-21

**Authors:** Theresa Vasko, Joana Frobel, Richard Lubberich, Tamme W. Goecke, Wolfgang Wagner

**Affiliations:** Helmholtz-Institute for Biomedical Engineering, Stem Cell Biology and Cellular Engineering, RWTH Aachen University Medical School, Pauwelsstrasse 20, 52074 Aachen, Germany; Institute for Biomedical Engineering—Cell Biology, University Hospital of RWTH Aachen, Aachen, Germany; Department of Obstetrics and Gynecology, RWTH Aachen University Hospital, Aachen, Germany

## Abstract

In vitro culture of hematopoietic stem and progenitor cells (HPCs) is supported by a suitable cellular microenvironment, such as mesenchymal stromal cells (MSCs)—but MSCs are heterogeneous and poorly defined. In this study, we analyzed whether MSCs derived from induced pluripotent stem cells (iPS-MSCs) provide a suitable cellular feeder layer too. iPS-MSCs clearly supported proliferation of HPCs, maintenance of a primitive immunophenotype (CD34^+^, CD133^+^, CD38^-^), and colony-forming unit (CFU) potential of CD34^+^ HPCs. However, particularly long-term culture-initiating cell (LTC-IC) frequency was lower with iPS-MSCs as compared to primary MSCs. Relevant genes for cell-cell interaction were overall expressed at similar level in MSCs and iPS-MSCs, whereas *VCAM1* was less expressed in the latter. In conclusion, our iPS-MSCs support in vitro culture of HPCs; however, under the current differentiation and culture conditions, they are less suitable than primary MSCs from bone marrow.

**Electronic supplementary material:**

The online version of this article (doi:10.1186/s13045-016-0273-2) contains supplementary material, which is available to authorized users.

We followed the hypothesis that iPS-MSCs might provide an unlimited and more standardized alternative to primary MSCs for stromal support of hematopoietic stem and progenitor cells (HPCs). To this end, we have reprogrammed bone marrow-derived MSCs into iPSCs and subsequently re-differentiated them towards iPS-MSCs as described before [1]. iPS-MSCs revealed similar fibroblastoid morphology, immunophenotype, and in vitro differentiation potential as primary MSCs (Additional file 1). HPCs were isolated from cord blood after written consent (Ethic Committee of RWTH Aachen: EK187/08). CD34^+^ cells were stained with carboxyfluorescein succinimidyl ester (CFSE) to monitor cell proliferation [2]. Flow cytometric analysis of residual CFSE staining after 5 days demonstrated that HPCs proliferated significantly faster if cultured with stromal support of either MSCs or iPS-MSCs (Fig. 1a). CD34 expression declined within a few cell divisions without feeder layer, whereas it was largely maintained over five subsequent cell divisions under both co-culture conditions (Fig. 1b). Overall, the expression of CD34 and CD133 declines after five cell divisions, which is consistent with previous observations [2]. Statistical analysis of CD34, CD38, CD45, and CD133 expression in relation to the cell division numbers indicated that co-culture with primary MSCs was slightly advantageous as compared to iPS-MSCs for maintenance of a primitive hematopoietic immunophenotype (Fig. 1c).

**Fig. 1:**
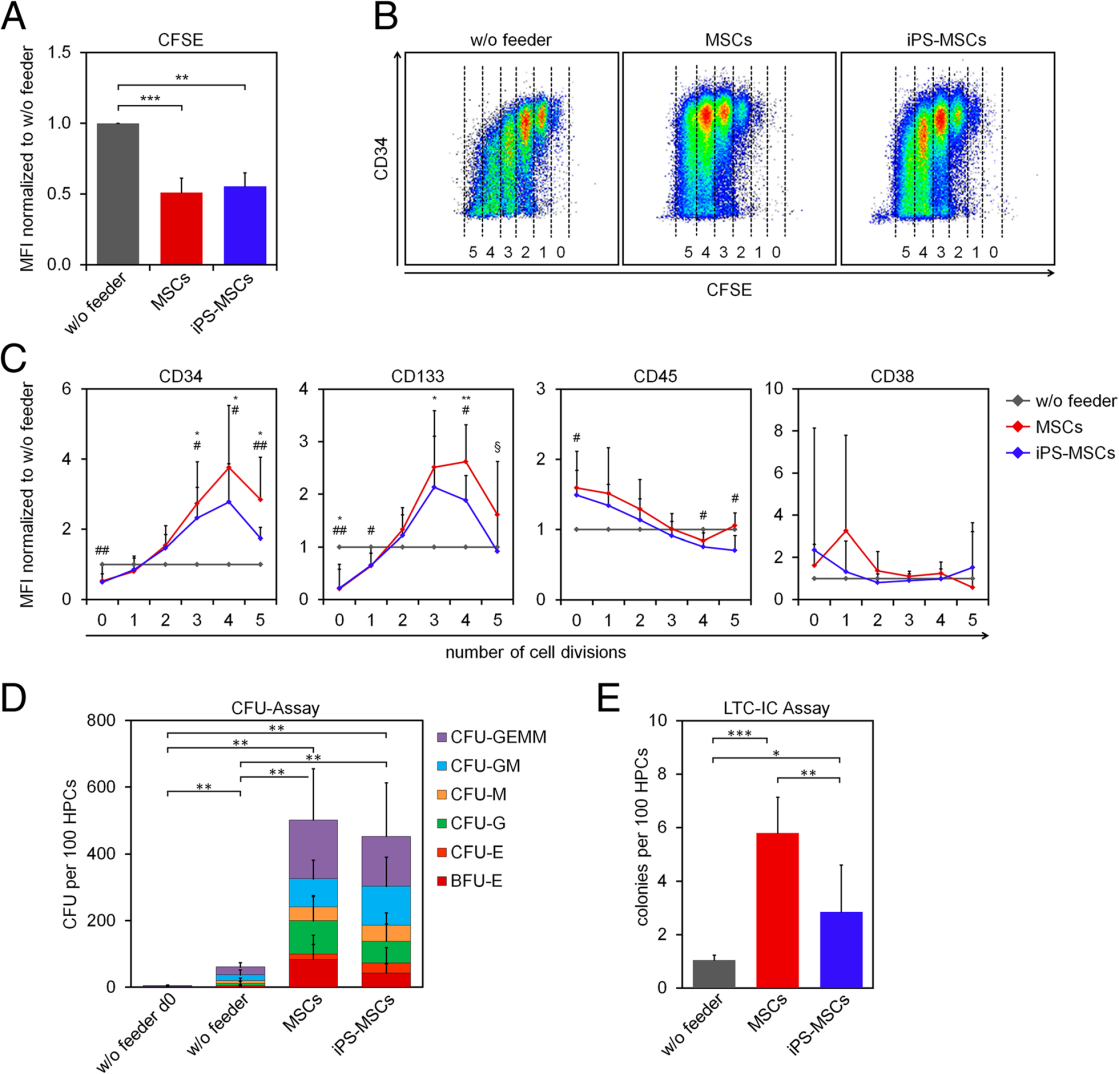
The hematopoietic supportive function of iPS-MSCs. **a** CD34^+^ cells were stained with CFSE and cultured with or without feeder cells for 5 days. Co-culture of HPCs with either MSCs or iPS-MSCs enhanced the number of cell divisions significantly (**P < 0.01; ***P < 0.001; *n* = 5—each with three biological replicates for MSCs and iPS-MSCs; MFI = mean fluorescence intensity). **b** Dot plots show CD34 expression in relation to the number of cell divisions (as a reference we used additional measurements at day 0; the number of cell divisions in different CFSE-gates is indicated). **c** We gated for specific cell division numbers and analyzed the signal intensity of CD34, CD133, CD45, and CD38 as compared to culture without feeder layer (*gray*). Co-culture with MSCs (*red*) or iPS-MSCs (*blue*) led to an increase of CD34 and CD133 expression and a decrease of CD45 expression in proliferating cells (without feeder vs. MSCs: *P < 0.05, **P < 0.01; without feeder vs. iPS-MSCs: ^#^ *P* < 0.05, ^##^ *P* < 0.01; MSCs vs. iPS-MSCs : ^§^ *P* < 0.05; *n* = 5—each with three biological replicates for MSCs and iPS-MSCs). **d** CFU frequency was significantly increased by co-culture with either MSCs or iPS-MSCs (**P < 0.01, *n* = 3—each with three biological replicates for MSCs and iPS-MSCs). There was no significant bias for specific types of colonies. BFU-E = burst-forming-unit erythroid; CFU-E = colony-forming-unit erythroid; CFU-G = colony-forming-unit granulocyte; CFU-M = colony-forming-unit macrophage; CFU-GM = colony-forming-unit granulocyte, macrophage; CFU-GEMM = colony-forming-unit granulocyte, erythrocyte, macrophage, megakaryocyte. **e** Frequency of long-term culture initiating cells (LTC-IC) was significantly higher in co-culture with primary bone marrow-derived MSCs as compared to iPS-MSCs or without stromal support (*P < 0.05, **P < 0.01, ***P < 0.001, *n* = 3—each with three biological replicates for MSCs and iPS-MSCs). Mean ± S.D. is depicted

We assessed the CFU frequency in freshly isolated HPCs or upon culture-expansion for 7 days: without stromal support, there was no expansion of CFUs, whereas CFU frequency was significantly increased under co-culture conditions with MSCs or iPS-MSCs (Fig. 1d). CFU frequency was not significantly affected if HPCs were co-cultured either with MSCs or iPS-MSCs, and there was no bias towards specific types of colonies (Fig. 1d). However, if HPCs were cultured for 5 weeks in a long-term culture-initiating cell (LTC-IC) assay [2], different hematopoiesis supporting capacities of MSCs and iPS-MSCs became evident: long-term culture of HPCs gave rise to a significantly higher number of colonies on MSCs compared to iPS-MSCs (Fig. 1e).

There is evidence that besides cytokine secretion, direct cell-cell interaction between HPCs and MSCs is crucial for the hematopoiesis supportive function and migration [3–5]—and this is reflected by cellular polarization [6, 7]. In fact, co-culture with MSCs gave rise to a significantly higher fraction of elongated cells as compared to iPS-MSCs or feeder-free conditions (Fig. 2a). Subsequently, we reanalyzed previously published gene expression profiles of MSCs, iPSCs, and iPS-MSCs (GSE46019, GSE38806, and GSE54766) [1] with focus on a set of genes that has been considered to be functionally relevant for cell-cell interaction [8]. Overall, these genes were expressed at very similar levels in MSCs and iPS-MSCs, underlining the close molecular relationship of both cell preparations (Fig. 2b). Among the selected genes, only laminin β1 (*LAMB1*) was higher expressed in iPS-MSCs (limma adjusted *P* value: *P* = 0.004), and the vascular cell adhesion molecule 1 (*VCAM1*; CD106) was higher expressed in MSCs (*P* = 0.0018). This trend was also observed by flow cytometric analysis of VCAM1 (Fig. 2c), although it was only expressed in a relatively small subset of MSCs. We have previously demonstrated that *VCAM1* is higher expressed in bone marrow-derived MSCs than in adipose tissue-derived MSCs [9]. Furthermore, we have shown that siRNA-mediated knockdown of *VCAM1* in MSCs entails lower proliferation rates of co-cultured HPCs [2]. It has been suggested that VCAM1 positive and negative subsets of MSCs differ in their biological function [10, 11] and that particularly the VCAM1 positive subset has higher immunoregulatory potential [11]. Lower expression of *VCAM1* in iPS-MSCs might therefore be one reason for reduced stromal support. On the other hand, N-cadherin (*CDH2*), which is also relevant for interaction of MSCs with HSCs [5], was in tendency higher expressed in iPS-MSCs than MSCs. Notably, differential expression of *LAMB1*, *VCAM1*, and *CDH2* was also reflected in DNA methylation patterns of MSCs and iPS-MSCs (Additional file 2). Either way, it is likely that a combination of adhesion proteins and chemokines evokes the differences in hematopoiesis supportive potential.

**Fig. 2:**
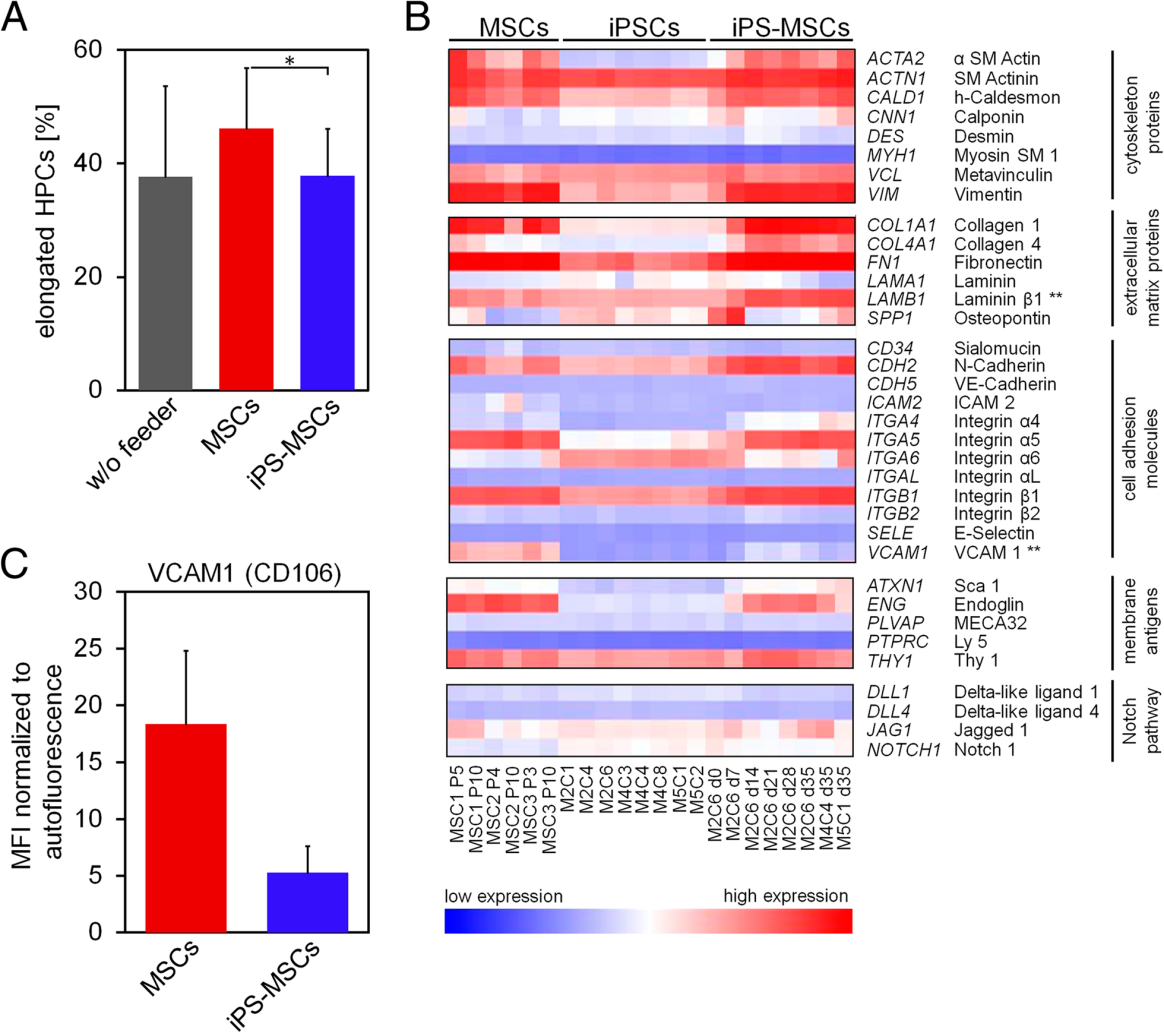
Differences in cell-cell interaction with different feeder layers. **a** The percentage of HPCs with elongated morphology was scored as described before [6]. Particularly, co-culture with MSCs stimulated cellular elongation (*P < 0.05, *n* = 3—each with three biological replicates for MSCs and iPS-MSCs). **b** Gene expression of relevant genes for cellular interaction with HPCs were analyzed in MSCs, iPSCs, and iPS-MSCs. Overall, all genes were expressed at very similar levels in primary MSCs and iPS-MSCs—except for Laminin β1 (*LAMB1*; higher expressed in iPS-MSCs; **P < 0.01) and vascular cell adhesion molecule 1 (*VCAM1*; higher expressed in MSCs; **P < 0.01). **c** Mean fluorescence intensity of VCAM1 (CD106) expression in flow cytometric analysis (three biological replicates for MSCs and iPS-MSCs). Mean ± S.D. is depicted

Taken together, our iPS-MSCs provide a less hematopoiesis supportive microenvironment than primary MSCs, particularly after long-term co-culture. This tendency was not observed in a recent study by Moslem et al. [12], but these authors did not test for maintenance of LTC-ICs. It is conceivable that optimized differentiation procedures of iPSCs towards cellular components of the hematopoietic stem cell niche as well as 3D-culture systems will further enhance stromal support to ultimately facilitate in vitro expansion of HPCs.

## Additional files

Additional file 1: Functional characterization of iPS-MSCs. (A) Phase contrast images of MSCs and iPS-MSCs: iPS-MSCs revealed similar fibroblastoid morphology as MSCs. (B) iPS-MSCs displayed similar immunophenotypic characteristics as primary MSCs (MFI = mean fluorescence intensity; mean ± S.D. of three biological replicates is presented; **P* < 0.05, ***P* < 0.01). (C) MSCs and iPS-MSCs were differentiated for three weeks towards adipogenic, osteogenic, and chondrogenic lineages and subsequently stained with BODIPY/DAPI, Alizarin Red, or Alcian Blue/PAS, respectively (in analogy to our previous work [1]). Controls were simultaneously cultured in normal growth medium (DMEM supplemented with 10 % human platelet lysate). Representative images are shown. (PDF 153 kb) Additional file 2: DNA methylation is in line with differential expression of *VCAM1*, *CDH2*, and *LAMB1*. DNA methylation levels of CpG dinucleotides in the genes *VCAM1*, *CDH2*, and *LAMB1* were analyzed for bone marrow-derived MSCs, iPS-MSCs, and iPSCs using the Illumina 450 k BeadChip data (GSE17448 and GSE54767) as described in detail in our previous work [1]. DNA methylation level is given as β-value ranging from 0 (no methylation) to 1 (100 % methylation). Genomic location of the respective CpG sites and statistical significance of MSCs vs. iPS-MSCs are indicated (**P* < 0.05, ***P* < 0.01, ****P* < 0.001, TSS1500 = 1500 bp upstream of transcription start site; TSS200 = 200 bp upstream of TSS; UTR = untranslated region). DNA methylation of *VCAM1* was higher in iPS-MSCs than MSCs. In contrast, close to the transcription start site of *LAMB1* and *CDH2* several CpGs revealed significantly lower DNA methylation in iPS-MSCs than primary MSCs. These epigenetic differences may therefore be relevant for the observed differences in gene expression. (PDF 362 kb)
