# Supplementary material for: iPSC-derived mesenchymal stromal cells are less supportive than primary MSCs for co-culture of hematopoietic progenitor cells
Source: J Hematol Oncol. 2016 Apr 21;9:43. doi: 10.1186/s13045-016-0273-2 (PMC4839158; doi:10.1186/s13045-016-0273-2)
Supplement: Additional file 1: — Functional characterization of iPS-MSCs. (A) Phase contrast images of MSCs and iPS-MSCs: iPS-MSCs revealed similar fibroblastoid morphology as MSCs. (B) iPS-MSCs displayed similar immunophenotypic characteristics as primary MSCs (MFI = mean fluorescence intensity; mean ± S.D. of three biological replicates is presented; *P < 0.05, **P < 0.01). (C) MSCs and iPS-MSCs were differentiated for three weeks towards adipogenic, osteogenic, and chondrogenic lineages and subsequently stained with BODIPY/DAPI, Alizarin Red, or Alcian Blue/PAS, respectively (in analogy to our previous work [1]). Controls were simultaneously cultured in normal growth medium (DMEM supplemented with 10 % human platelet lysate). Representative images are shown. (PDF 153 kb) [file 13045_2016_273_MOESM1_ESM.pdf]

Additional file 1

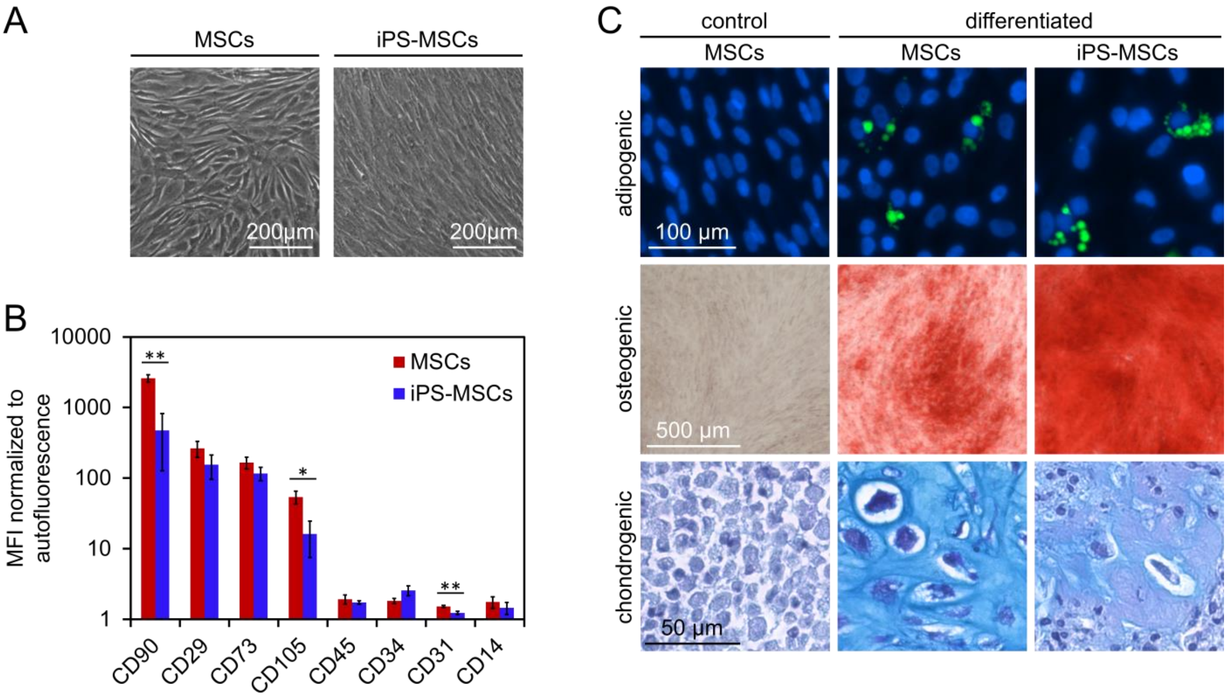

Additional Figure 1. Functional characterization of iPS-MSCs.

(**A**) Phase contrast images of MSCs and iPS-MSCs: iPS-MSCs revealed similar fibroblastoid morphology as MSCs. (**B**) iPS-MSCs displayed similar immunophenotypic characteristics as primary MSCs (MFI = mean fluorescence intensity; mean ± S.D. of three biological replicates is presented; *$P < 0.05$, **$P < 0.01$). (**C**) MSCs and iPS-MSCs were differentiated for three weeks towards adipogenic, osteogenic, and chondrogenic lineages and subsequently stained with BODIPY/DAPI, Alizarin Red, or Alcian Blue/PAS, respectively (in analogy to our previous work [1]). Controls were simultaneously cultured in normal growth medium (DMEM supplemented with 10% human platelet lysate). Representative images are shown.
