# Supplementary material for: iPSC-derived mesenchymal stromal cells are less supportive than primary MSCs for co-culture of hematopoietic progenitor cells
Source: J Hematol Oncol. 2016 Apr 21;9:43. doi: 10.1186/s13045-016-0273-2 (PMC4839158; doi:10.1186/s13045-016-0273-2)
Supplement: Additional file 2: — DNA methylation is in line with differential expression of VCAM1, CDH2, and LAMB1. DNA methylation levels of CpG dinucleotides in the genes VCAM1, CDH2, and LAMB1 were analyzed for bone marrow-derived MSCs, iPS-MSCs, and iPSCs using the Illumina 450 k BeadChip data (GSE17448 and GSE54767) as described in detail in our previous work [1]. DNA methylation level is given as β-value ranging from 0 (no methylation) to 1 (100 % methylation). Genomic location of the respective CpG sites and statistical significance of MSCs vs. iPS-MSCs are indicated (*P < 0.05, **P < 0.01, ***P < 0.001, TSS1500 = 1500 bp upstream of transcription start site; TSS200 = 200 bp upstream of TSS; UTR = untranslated region). DNA methylation of VCAM1 was higher in iPS-MSCs than MSCs. In contrast, close to the transcription start site of LAMB1 and CDH2 several CpGs revealed significantly lower DNA methylation in iPS-MSCs than primary MSCs. These epigenetic differences may therefore be relevant for the observed differences in gene expression. (PDF 362 kb) [file 13045_2016_273_MOESM2_ESM.pdf]

Additional file 2

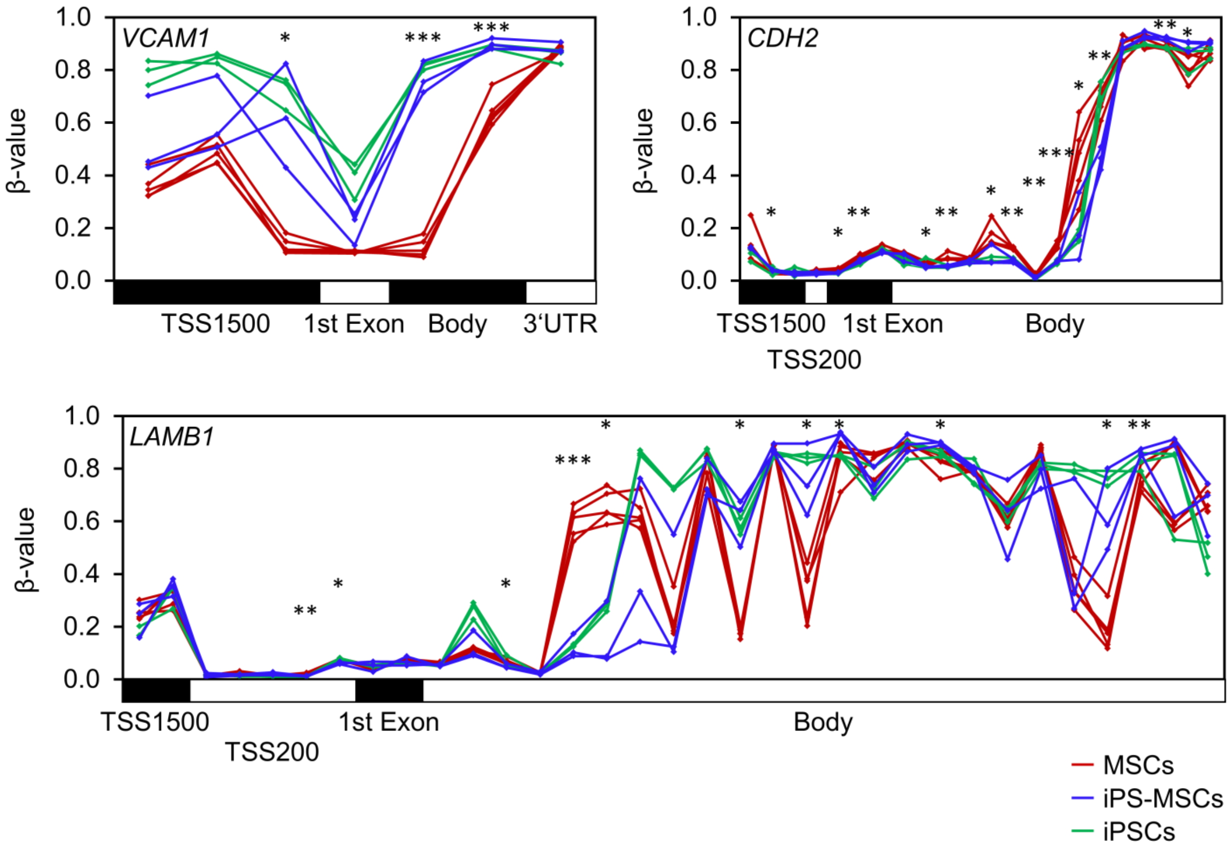

Additional Figure 2. DNA methylation is in line with differential expression of *VCAM1*, *CDH2*, and *LAMB1*. DNA methylation levels of CpG dinucleotides in the genes *VCAM1*, *CDH2*, and *LAMB1* were analyzed for bone marrow-derived MSCs, iPS-MSCs, and iPSCs using the Illumina 450k BeadChip data (GSE17448 and GSE54767) as described in detail in our previous work [1]. DNA methylation level is given as β-value ranging from 0 (no methylation) to 1 (100% methylation). Genomic location of the respective CpG sites and statistical significance of MSCs vs. iPS-MSCs are indicated (**P* < 0.05, ***P* < 0.01, ****P* < 0.001, TSS1500 = 1,500 bp upstream of transcription start site; TSS200 = 200 bp upstream of TSS; UTR = untranslated region). DNA methylation of *VCAM1* was higher in iPS-MSCs than MSCs. In contrast, close to the transcription start site of *LAMB1* and *CDH2* several CpGs revealed significantly lower DNA methylation in iPS-MSCs than primary MSCs. These epigenetic differences may therefore be relevant for the observed differences in gene expression.
